# Right ventricular assessment with cardiac magnetic resonance: usefulness in routine clinical practice compared to echocardiography

**DOI:** 10.1186/1532-429X-11-S1-P114

**Published:** 2009-01-28

**Authors:** Covadonga Fernández-Golfín, José Zamorano Gómez, Cecilia Corros Vicente, Tibisay Sánchez, Joaquin Ferreiros, Leopoldo Pérez de Isla, Ana Bustos, Betariz Cabeza, Carlos Macaya

**Affiliations:** Hospital Universitario Clinico San Carlos, Madrid, Spain

**Keywords:** Right Ventricular, Cardiac Magnetic Resonance, Right Ventricular Function, Right Ventricular Dysfunction, Right Ventricular Ejection Fraction

## Introduction

Right ventricular (RV) morphology and function is essential in the evaluation of different cardiac diseases. It has prognosis significance and influence everyday clinical decision making. Routine RV assessment is done by means of echocardiography. Visual estimation of RV morphology and function is normally performed, using RV function echocardiographic indexes only in selected populations. This approach may lead to inaccurate RV assessment especially in cases with mild RV dilatation/impairment. Cardiac magnetic resonance (CMR) has emerged as the gold standard for biventricular volume and function quantiphication.

## Purpose

The aim of the study was to analyze in a clinical setting, the utility of CMR in the evaluation of RV morphology and function, comparing it to routine echocardiography examinations reports.

## Methods

96 patients with RV volume and function assessed with CRM were initially included. Reports form echocardiography performed to these patients were reviewed. Patients in whom the echocardiography was performed more than one year from the CRM study were excluded, as well as patients in whom specific echocardiography RV information was lacking. Study population consisted of 72 patients, mean age 48 years, men 55, 6%. Main indication for CRM study was as follows: viability assessment 7% dilated cardyomyopathy 11, 1% and right ventricular dysplasia evaluation 7%. All CMR exams were performed at 1,5 T (Signa, General Electrics, Milwaukee, WI) scanner. Breath-hold, electrocardiographically triggered steady state free precession cine images were acquired in four chamber long axis view and short axis view from base to apex. Cine images were analyzed with CMR tools (Cardiac Report Card 2.0, General Electrics). RV end diastolic, end systolic volume and ejection fraction were calculated using Simpson method. Volumes and ejection fraction were then classified as normal/abnormal in accordance to patient age and sex using published reference values. Based on echocardiography reports RV was considered dilated or not and RV ejection fraction normal or decreased in a binary manner. Statistical analysis was performed using SPSS program, version 12.

## Results

72 patients were included. Mean RVEDV was 133,21 ml, RVESV 56,68 ml and RVEF 58,94. According to CRM 11, 1% patients had increased end diastolic RVV with 25% having increased end systolic RVV. Right ventricular dysfunction was present in 25%. Echocardiography studies described RV dilatation in 15, 5 patients and RV dysfunction in 5, 6% patients. Agreement index between both methods were 0,33 (p 0,004) for RV volume and 0,19 (p 0,019) for RV ejection fraction.

## Conclusion

Routine echocardiography evaluation of RV in different cardiac diseases compared to gold standard CRM lack accuracy. Thus, in cases where RV assessment is essential other echo Doppler indexes or CRM should be considered in order to provide accurate RV evaluation.

